# Saudi Oncology Society clinical management guidelines for urinary bladder cancer

**DOI:** 10.4103/0974-7796.78549

**Published:** 2011-03

**Authors:** Khaled Al Othman, Shouki Bazarbashi, Khalid Balaraj, Mohamed Al Otaibi, Baher Kamal, Ibraheem Al Oraifi, Eyad Al Saeed, Khalid Al Gamdi, Ali Jubran, Ahmad Salah, Jalal Al Shareef, Jamal Zekri

**Affiliations:** Department of Urology, King Faisal Specialist Hospital and Research Centre, Riyadh, Saudi Arabia

**Keywords:** Urinary bladder cancer, Saudi, guidelines

## Abstract

In this report guidelines for the evaluation, medical and surgical management of transitional cell carcinoma of urinary bladder is presented. It is categorized according to the stage of the disease using the tumor node metastasis staging system, 7^th^ edition. The recommendations are presented with supporting level of evidence.

## INTRODUCTION

There were 211 cases of bladder cancer accounting for 2.6% of all newly diagnosed cases in the year 2006 in Saudi Arabia. This cancer ranked ninth among male population and twentieth among female population. It affected 169 (80.1%) males and 42 (19.9%) females with a male to female ratio of 4:1. The overall ASR was 2.6/100,000; 4.1/100,000 for males and 1/100,000 for female.[1]

## 1. STAGING[2]

See Appendix I

## 2. GRADING

The World Health Organization (WHO) grading of urinary tumors 2004[3] will be used as follows:

**Table:** 

2.1.	Urothelial papilloma
2.2.	PUNLMP: Papillary urothelial neoplasm of low malignant potential
2.3.	Low-grade papillary urothelial carcinoma
2.4.	High-grade papillary urothelial carcinoma

## 3. NON-MUSCLE INVASIVE BLADDER CANCER (TA, T1, TIS)

**Table:** 

3.1.	Evaluation should include:
3.1.1.	History and physical examination
3.1.2.	Imaging:
3.1.2.1.	For non-muscle invasive tumors, imaging of upper urinary tract (CT or IVU) is indicated if patient has tumors located in the trigone, multifocal or high-risk tumors (see item 3.2.3).[4,5] (EL3)
3.1.2.2.	CT abdomen/pelvis or MRI and CXR or CT chest is indicated for staging of muscle invasive bladder tumor. (EL3)
3.1.3.	Urine cytology
3.1.4.	Cystoscopy, which should include:
3.1.4.1.	Transurethral resection of bladder tumors (TURBT): The following should be observed:
3.1.4.1.1.	The goal of TURBT is to define the stage and grade of tumor (diagnostic) and to resect all grossly visible tumors (therapeutic).
3.1.4.1.2.	Deep resection is important to assess the depth of tumor invasion to the muscle.
3.1.4.1.3.	Random bladder and prostatic urethral biopsies are indicated only in patients with positive urine cytology with normal appearing bladder.[6–8] (EL3)
3.1.4.1.4.	Second TURBT is recommended to be done within 2–6 weeks from initial resection in the following conditions:[9–11] (EL2)
3.1.4.1.4.1.	Incomplete initial resection
3.1.4.1.4.2.	No muscle tissue in initial resection specimen
3.1.4.1.4.3.	High-grade NMIBT (Non-muscle invasive bladder tumor)
3.1.4.1.4.4.	T1 bladder tumor
3.2.	Risk stratification for non-muscle invasive bladder cancer: This depends on the following factors: tumor stage, grade, presence of carcinoma *in situ*, number of tumors, tumor size and prior recurrence rate:[12]
3.2.1.	Low-risk NMIBC (small volume, low-grade Ta)
3.2.2.	Intermediate risk NMIBC (multifocal and/or large-volume low-grade Ta, recurrence at 3 months)
3.2.3.	High-risk NMIBC (high-grade Ta, all T1, CIS)
3.3.	Intravesical therapy:
3.3.1.	Low-risk tumors: A single immediate post-operative instillation of mitomycin C or doxorubicin within 24 h (preferably within 6 h) if no suspicion of bladder perforation should be considered.[13] (EL1)
3.3.2.	Intermediate risk: it is recommended to give single immediate instillation of chemotherapy followed by induction BCG.[14] (EL2)
3.3.3.	High risk
3.3.3.1.	Carcinoma *in situ:*
3.3.3.1.1.	It is recommended to give induction intravesical BCG plus maintenance for at least 1 year.[15,16] (EL1)
3.3.3.1.2.	Assess response at 3 months, if no response:
3.3.3.1.2.1.	Additional 6 weeks course of BCG or
3.3.3.1.2.2.	Radical cystectomy or
3.3.3.1.2.3.	If no complete response at 6 months, radical cystectomy.[17]
3.3.3.2.	Multiple high-grade Ta–T1:
3.3.3.2.1.	It is recommended to repeat TURBT at 2–6 weeks, after initial resection.
3.3.3.2.2.	Intravesical BCG induction plus maintenance for at least 1 year.
3.3.3.2.3.	Immediate radical cystectomy can be considered for highest risk patients (T1 high grade with or without CIS)[18]. (EL3)
3.4.	Treatment of intravesical therapy failure:
3.4.1.	Definition of intravesical therapy failure:[18]
3.4.1.1.	Whenever muscle invasion is detected during follow up.
3.4.1.2.	If high-grade non-muscle invasive bladder cancer is present at 3 or 6 months.
3.4.1.3.	Any worsening of the disease with BCG treatment like higher stage or grade or appearance of CIS.
3.4.2.	Management of intravesical therapy failure:
3.4.2.1.	Patients with recurrence of NMIBC following immediate intravesical chemotherapy may benefit from BCG treatment.
3.4.2.2.	Patients with initial BCG therapy failure who experience recurrence of high-grade disease at 6 months should be offered cystectomy.[19]
3.4.2.3.	In case of failure before maintenance BCG has been completed, cystectomy should be considered if high-grade T1 or CIS is present. But for high-grade Ta recurrences, repeat resection and induction intravesical therapy could be started.[20] (EL3)
3.5.	Follow-up:
3.5.1.	Low risk: Cystoscopy and cytology at 3 months - if negative, next cystoscopy and cytology at 12 months and then yearly for 5 years. (EL3)
3.5.2.	High risk: Cystoscopy and cytology at 3 months, if negative, following cystoscopies should be repeated every 3 months for 2 years, at every 4 months in the third year and then every 6 months until 5 years and annually thereafter.
3.5.3.	Intermediate risk: Similar to high risk, however schedule can be adapted according to individual patient.[18]
3.5.4.	Annual imaging of upper urinary tract in high-risk group

## 4. MUSCLE INVASIVE BLADDER CANCER: OPTIONS INCLUDE

**Table:** 

4.1.	Radical cystectomy and urinary diversion:
4.1.1.	Radical cystectomy is the preferred curative treatment for localized bladder cancer (EL3)
4.1.2.	Radical cystectomy includes removal of regional lymph nodes, the extent of which has not been sufficiently defined (EL3)
4.1.3.	Laparoscopic and robotic radical cystectomy are optional
4.1.4.	An orthotopic bladder substitute option should be offered to male and female patients lacking any contra-indications.
4.1.5.	Neoadjuvant cisplatin-based chemotherapy improved overall survival by 5–7% at 5 years and this option should be offered to patients especially with locally advanced disease (T3,T4).[21–23] (EL1)
4.1.6.	Follow-up after radical cystectomy:
4.1.6.1.	Urine cytology, creatinine, electrolytes, every 3 to 9 months for 2 years and then as clinically indicated.[24]
4.1.6.2.	CT chest, abdomen and pelvis every 3 to 9 months for 2 years based on risk of recurrence and as clinically indicated.
4.1.6.3.	Urethral wash cytology, every 6 to 12 months. (EL3)
4.2.	Radiation therapy should be offered for patients with localized disease not fit for surgery and chemotherapy. (EL3)
4.3.	Bladder sparing treatment: multimodality treatment should be considered as an option for selected group of patients and well-informed compliant patients (EL3):
4.3.1.	Patients selected for bladder sparing treatment should have the following:
4.3.1.1.	Clinically T2-T3 tumor
4.3.1.2.	No hydronephrosis
4.3.1.3.	Normal renal function
4.3.1.4.	No multifocal disease or carcinoma *in situ*
4.3.1.5.	Functional bladder
4.3.1.6.	Urothelial histology
4.3.1.7.	No prostatic urethral involvement
4.3.2.	Multimodality therapy should consist of:
4.3.2.1.	Aggressive and visibly complete TURBT.
4.3.2.2.	Concurrent cisplatin at 100 mg/m2 at day 1 and 22 of radiation therapy.
4.3.2.3.	Radiation therapy at 1.8 Gy/fraction
4.3.2.4.	Cystoscopy (within 2 weeks) after the initial phase (45 Gy): patients with positive biopsy or cytology should undergo radical cystectomy. Patients with negative results would continue radiation with a cone down beam for a total of 64.8 Gy and one more cycle of cisplatin.
4.3.3.	Follow up should include cystoscopy every 3 months for the first 2 years, then every 6 months for the next 3 years and then annually.
4.3.4.	Superficial recurrent disease should be treated locally (TURBT ± BCG). (EL3)
4.4.	Adjuvant chemotherapy
4.4.1.	Adjuvant chemotherapy could be considered using Cisplatin and gemcitabine regimen in patients with:[25]
4.4.1.1.	Normal renal function.
4.4.1.2.	Performance status 0-2.
4.4.1.3.	Pathological stage T3, 4 or node-positive disease.
4.4.1.4.	Patients should not have received neo-adjuvant chemotherapy.
4.4.1.5.	Urothelial histology

## 5. ADVANCED, METASTATIC AND RECURRENT DISEASE: CHEMOTHERAPY IS THE MAINSTAY OF THERAPY

**Table:** 

5.1.	Patients with normal renal function and fit for chemotherapy (PS 0–2), are treated with combination cisplatin and gemcitabine for a maximum of 6 cycles (EL1).[26]
5.2.	Patients with decreased renal function and / or unfit (PS 3) are treated with combination of Carboplatin and gemcitabine or single agent gemcitabine (EL2).[27]
5.3.	Patient who relapse or progress on the above regimens may be given taxanes as second-line chemotherapy (EL2).
5.4.	Patients who present with local recurrence may benefit from palliative radiation therapy

## APPENDIX I: TNM STAGING

**Table:**

Primary tumor (T)	
TX	Primary tumor cannot be assessed
T0	No evidence of primary tumor
Ta	Non-invasive papillary carcinoma
Tis	Carcinoma *in situ*: “flat tumor”
T1	Tumor invades subepithelial connective tissue
T2	Tumor invades muscularis propria
pT2a	Tumor invades superficial muscularis propria (inner half)
pT2b	Tumor invades deep muscularis propria (outer half)
T3	Tumor invades perivesical tissue
pT3a	Microscopically
pT3b	Macroscopically (extravesical mass)
T4	Tumor invades any of the following: prostatic stroma, seminal vesicles, uterus, vagina, pelvic wall, abdominal wall.
T4a	Tumor invades prostatic stroma, uterus, vagina
T4b	Tumor invades pelvic wall, abdominal wall
Regional lymph nodes (N)*	
NX	Lymph nodes cannot be assessed
N0	No lymph node metastasis
N1	Single regional lymph node metastasis in the true pelvic (hypogastric, obturator, external iliac or presacral lymph node)
N2	Multiple regional lymph node metastasis in the true pelvis (hypogastric, obturator, external iliac or presacral lymph node metastasis)
N3	Lymph node metastasis to the common iliac lymph nodes
Distant metastasis (M)	
M0	No distant metastasis
M1	Distant metastasis
Anatomic stage / prognostic groups	
Stage 0a	Ta	N0	M0
Stage 0is	Tis	No	M0
Stage I	T1	N0	M0
Stage II	T2a	N0	M0
	T2b	N0	M0
Stage III	T3a	N0	M0
	T3b	N0	M0
	T4a	N0	M0
Stage IV	T4b	N0	M0
	Any T	N1-3	M0
	Any T	Any N	M1
